# Evaluation of the diagnostic utility on 1.5T and 3.0T ^1^H magnetic resonance spectroscopy for temporal lobe epilepsy

**DOI:** 10.1186/s12880-023-01136-w

**Published:** 2023-11-14

**Authors:** Biao Qu, Hejuan Tan, Min Xiao, Dongbao Liu, Shijin Wang, Yiwen Zhang, Runhan Chen, Gaofeng Zheng, Yonggui Yang, Gen Yan, Xiaobo Qu

**Affiliations:** 1 Department of Instrumental and Electrical Engineering, Xiamen University, Xiamen, China; 2 Institute of Artificial Intelligence, Xiamen University, Xiamen, China; 3 Biomedical Intelligent Cloud R&D Center, Fujian Provincial Key Laboratory of Plasma and Magnetic Resonance, Department of Electronic Science, Xiamen University, Xiamen, China; 4 Department of Information & Computational Mathematics, Xiamen University, Xiamen, China; 5 Department of Neurology, The Second Affiliated Hospital of Xiamen Medical College, Xiamen, China; 6 National Institute for Data Science in Health and Medicine, Xiamen University, Xiamen, China; 7 Department of Radiology, The Second Affiliated Hospital of Xiamen Medical College, Xiamen, China

**Keywords:** Brain, ^1^H-MRS, Metabolites, Temporal lobe epileptic, 1.5T, 3.0T

## Abstract

**Background:**

^1^H magnetic resonance spectroscopy (^1^H-MRS) can be used to study neurological disorders because it can be utilized to examine the concentrations of related metabolites. However, the diagnostic utility of different field strengths for temporal lobe epilepsy (TLE) remains unclear. The purpose of this study is to make quantitative comparisons of metabolites of TLE at 1.5T and 3.0T and evaluate their efficacy.

**Methods:**

Our retrospective collections included the single-voxel ^1^H-MRS of 23 TLE patients and 17 healthy control volunteers (HCs) with a 1.5T scanner, as well as 29 TLE patients and 17 HCs with a 3.0T scanner. Particularly, HCs were involved both the scans with 1.5T and 3.0T scanners, respectively. The metabolites, including the N-acetylaspartate (NAA), creatine (Cr), and choline (Cho), were measured in the left or right temporal pole of brain. To analyze the ratio of brain metabolites, including NAA/Cr, NAA/Cho, NAA/(Cho + Cr) and Cho/Cr, four controlled experiments were designed to evaluate the diagnostic utility of TLE on 1.5T and 3.0T MRS, included: (1) 1.5T TLE group vs. 1.5T HCs by the Mann-Whitney U Test, (2) 3.0T TLE group vs. 3.0T HCs by the Mann-Whitney U Test, (3) the power analysis for the 1.5T and 3.0T scanner, and (4) 3.0T HCs vs. 1.5T HCs by Paired T-Test.

**Results:**

Three metabolite ratios (NAA/Cr, NAA/Cho, and NAA/(Cho + Cr) showed the same statistical difference (p < 0.05) in distinguishing the TLE from HCs in the bilateral temporal poles when using 1.5T or 3.0T scanners. Similarly, the power analysis demonstrated that four metabolite ratios (NAA/Cr, NAA/Cho, NAA/(Cho + Cr), Cho/Cr) had similar distinction abilities between 1.5T and 3.0T scanner, denoting both 1.5T and 3.0T scanners were provided with similar sensitivities and reproducibilities for metabolites detection. Moreover, the metabolite ratios of the same healthy volunteers were not statistically different between 1.5T and 3.0T scanners, except for NAA/Cho (p < 0.05).

**Conclusions:**

1.5T and 3.0T scanners may have comparable diagnostic potential when ^1^H-MRS was used to diagnose patients with TLE.

## Background

Epilepsy is a kind of serious neurological disorder of the brain [1]. The seizures of epilepsy can be divided into the types of focal, generalized and unknown [2]. As the most common form of focal seizures [3], temporal lobe epilepsy (TLE) approximately accounts for 60% of adult epilepsy cases [2, 4]. During this disease attack, a loss of consciousness, disturbances of limb movements, laloplegia like temporary alogia and other symptoms will occur. Nevertheless, the diagnosis of TLE is still challenging since some resources, including the gold standard, complete clinical history and reliable patient testimony are not accessible [1, 5]. Video electroencephalogram (V-EEG) and magnetic resonance imaging (MRI) are typical of current diagnosis techniques of TLE [5]. V-EEG can judge the possible type of TLE (focal seizures or generalized seizures) and assess the risk of recurrence by detecting abnormal patterns, but it is time-consuming in monitoring as the complete procedure may take more than 3 days on average [6]. MRI can identify the location of epilepsy lesions but roughly 30% of TLE patients have normal brain MRI results [7].

Studies have shown that TLE is associated with extensive neuronal dysfunction, which may be caused by brain damage or genetic mutations [8]. ^1^H magnetic resonance spectroscopy (^1^H-MRS) is an important non-invasive diagnostic tool for TLE. Brain metabolite concentrations can be measured by ^1^H-MRS, mainly including N-acetylaspartate (NAA), creatine (Cr), and choline (Cho) [9, 10]. NAA is synthesized in mitochondria [11] and serves as a crucial marker for neuronal impairment. Anomalies in neuronal structures, such as diminished neuronal viability, result in decreased level of NAA [12]. Cr is thought to be involved in the process of neuronal damage and alter neural metabolite levels in recurrent patient with TLE [13]. Stored in the cell membrane, Cho is necessary for all cells to function normally, affecting nerve signaling, cell signaling and lipid transport/metabolism [14]. Indications of abnormalities in the temporal lobe of TLE patients can be provided by these metabolite spectra, even if no abnormality is found on patients’ MRI images [15, 16]. Comparing with the contralateral hippocampus, NAA was less on the side of the affected temporal lobe in TLE patients’ hippocampi [17], and the concentration of NAA in the epileptogenic foci was lower than that in the non-epileptogenic regions [18] as well. Significantly high Cho/Cr ratios were observed in the right thalamus in the focal impaired awareness seizures [9, 19]. The NAA/Cr ratio proved to be a useful biomarker to discriminate TLE seizures from organic non-epileptic seizures [10]. A decline in the NAA/Cho ratio is another significant indicator for identifying the region of epilepsy [18].

These observations are based on the MRS data acquired from a sole magnetic resonance specific field strength, e.g. 1.5T or 3.0T. For instance, the research of epilepsy was conducted on 1.5T scanners [20, 21] while others were performed on 3.0T scanners [19, 22]. High magnetic field strength scanners can achieve a higher signal-to-noise ratio (SNR) and improve spectral dispersion. However, it is unclear whether ^1^H-MRS for TLE diagnosis can be affected by MRI scanners at different field strengths.

The purpose of this research is to study the differences in the diagnostic efficacy of ^1^H-MRS for TLE between 1.5T and 3.0T.

## Methods

### Participants and experimental design

**Fig. 1:**
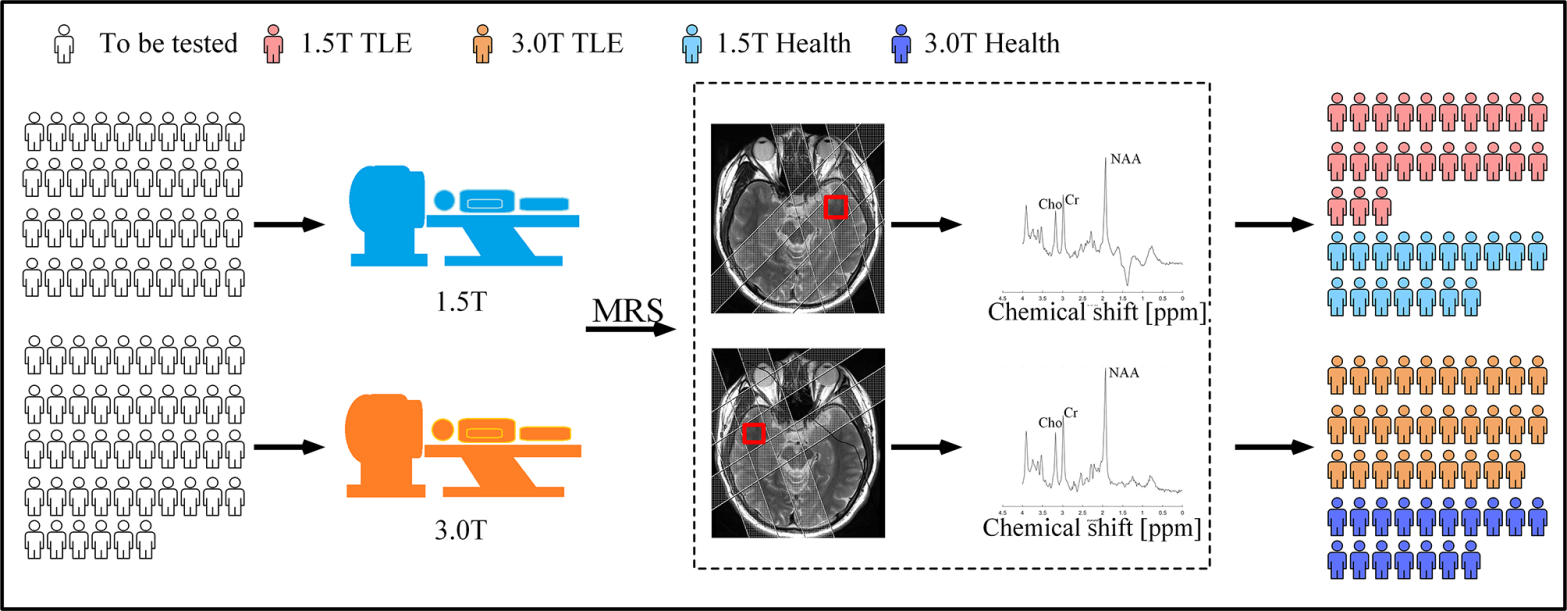
^1^H-MRS data collection flowchart of the study. The left and right temporal pole were used as regions of interest (ROI) and represented by red boxes to obtain metabolite concentrations of Cho, Cr, and NAA of subjects. The outer volume suppression (OVS), which are not parallel to the voxel edges, are manually added to enhance the saturation effect

This study was approved by the ethics committee of the Second Affiliated Hospital of Xiamen Medical College. Figure 1 shows the data collection process, we retrospectively collected single-voxel ^1^H-MRS of bilateral temporal poles from 2017 to 2021. There were 23 TLE patients diagnosed with the 1.5T scanner (15 males and 8 females, age: 29.52 ± 13 [Mean ± Standard Deviation (SD)]), 29 TLE patients diagnosed with the 3.0T scanner (20 males and 9 females, age: 28.2 ± 9.0), and 17 healthy controls (HCs) (11 males and 6 females, age: 23.35 ± 4.11) were scanned with both 1.5T and 3.0T MR scanners. To minimize the impact of multi-factor experimental conditions on outcomes, data were collected using scanners with different field strengths on the same healthy volunteer. To reduce the effect of time, HCs was scanned in batches in July 2021, with each batch scanned at both 1.5T and 3.0T in the same evening.

The diagnosis procedure of TLE seizures was as follows: First, the neurologist identifies whether the patient was having a seizure according to clinical symptoms, and then employed auxiliary monitoring, such as blood tests, neurological examinations, and electrocardiograms, to rule out causes of non-brain abnormalities; Second, TLE seizures were confirmed by recording epileptiform discharges through an electroencephalogram; Finally, by analyzing imaging (MRI and ^1^H-MRS) and genes, neuroradiologists ascertained the cause of temporal lobe epileptic seizures (TLES). The whole process was completed by a neuroradiologist and an experienced neurologist, and the diagnosis result was finally given by an authoritative neurologist.

**Fig. 2:**
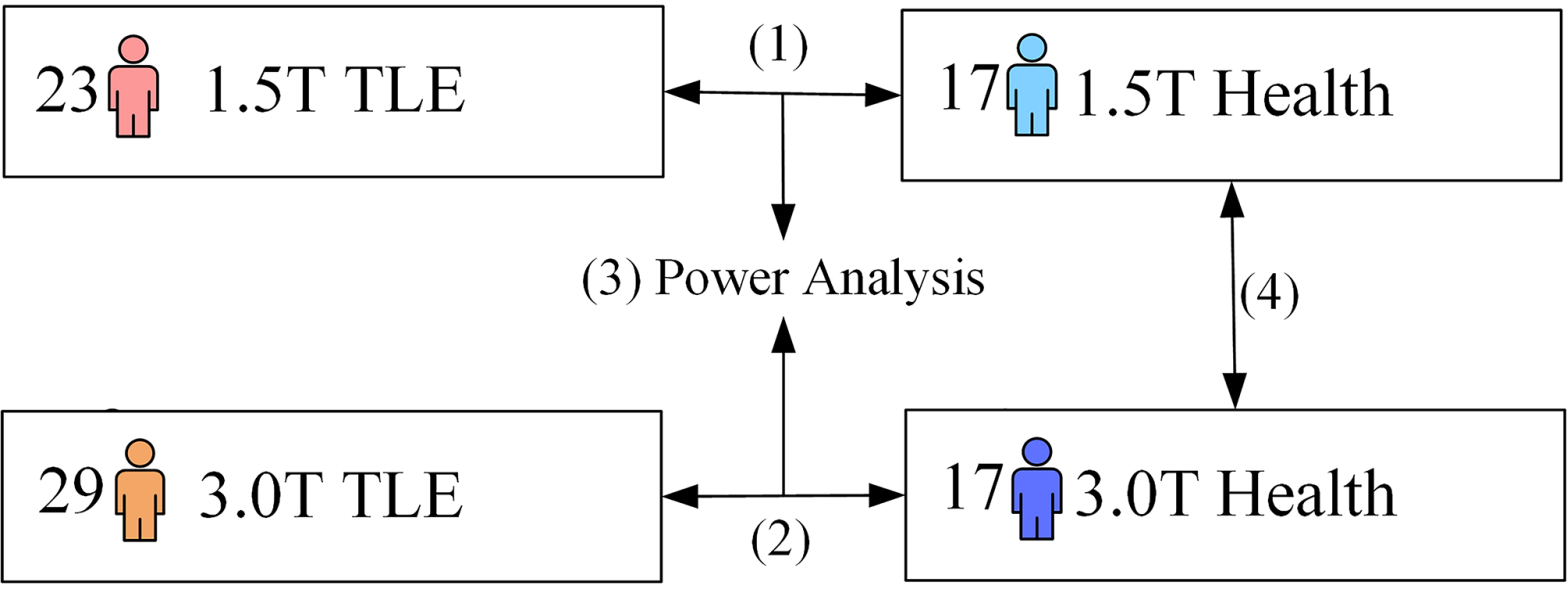
Schematic diagram of four control experiments. The number on the left side of the box indicates the sample size in an experiment

The ^1^H-MRS data were acquired from the bilateral temporal poles of all subjects, and four control experiments were designed (Fig. 2) as followed: (1) 1.5T TLE group vs. 1.5T HCs; (2) 3.0T TLE group vs. 3.0T HCs; (3) the power analysis between the 3.0T scanner and 1.5T scanner based on the statistical test of the TLE and HCs; and (4) 3.0T HCs vs. 1.5T HCs. These comparisons were aimed at evaluating the differences in the diagnostic utility of TLE at 1.5T and 3.0T, both of which were the most widespread magnetic resonance fields.

### MRI and ^1^H-MRS acquisition

#### MRI acquisition parameters

1.5T MR scanner was equipped with GE SIGNA HD medical system, and the specific imaging parameters were as follows: (1) T2 FLAIR sequence: TR = 8600ms, TE = 120ms, TI = 2100ms, FOV = 240 × 240mm^2^, matrix = 288 × 160, slice thickness = 5 mm, NEX = 1; (2) FRFSE T2WI sequence: TR = 4760ms, TE = 102ms, FOV = 240 × 240mm^2^, matrix = 320 × 256, slice thickness = 5 mm, number of slices = 19, NEX = 1; (3) DWI: b value = 1000s/mm^2^, TR = 6000ms, TE = 56ms, FOV = 240 × 240mm^2^, matrix = 128 × 128, slice thickness = 5 mm, number of slices = 19, NEX = 2.

3.0T MR scanner was equipped with GE Discovery Silent MR (750 W) medical system, and the specific imaging parameters were as follows: (1) T2 FLAIR sequence: TR = 8600ms, TE = 140ms, TI = 2100ms, FOV = 240 × 240mm^2^, matrix = 288 × 224, slice thickness = 5 mm, number of slices = 22, NEX = 1; (2) T2WI: TR = 4425ms, TE = 90ms, FOV = 240 × 240mm^2^, matrix = 384 × 384, slice thickness = 5 mm, number of slices = 22; NEX = 1, (3) DWI: b value = 1000s/mm^2^, TR = 4250ms, TE = 56ms, FOV = 240 × 240mm^2^, matrix = 128 × 128, slice thickness = 5 mm, number of slices = 22, NEX = 2.

#### ^1^H-MRS acquisition parameters

As shown in Table 1, PRESS sequence was applied to 1.5T scanner with the following sequence parameters: voxel size = 20 × 20 × 20mm^3^, chemical shift imaging layer thickness = 20 mm, NEX = 128, TR = 2000ms, TE = 144ms. PRESS sequence was also applied to 3.0T scanner with the following sequence parameters: voxel size = 20 × 20 × 20mm^3^, chemical shift imaging layer thickness = 20 mm, NEX = 128, TR = 1500ms, TE = 144ms. High-resolution T2W was used to localize single voxels in regions of interest (ROI) in the temporal lobe that coincides with the seizure onset zone determined by electroencephalography (EEG).

**Table 1:** The settings of the PRESS sequence parameters at 1.5T and 3.0T

Field strength	1.5T	3.0T
Voxel size	20 × 20 × 20mm^3^	20 × 20 × 20mm^3^
NEX	128	128
TR/TE	2000/144ms	1500/144ms

### Data pre-processing

After the MRS data of bilateral temporal lobes are collected, technicians use the built-in MRS software package (SAGE7.1) of GE scanner to quantify the data with the default mode to obtain the metabolite concentrations, and then calculate the metabolite concentration ratios, including NAA/Cr, NAA/Cho, NAA/(Cho + Cr) and Cho/Cr.

### Statistical analysis

The variables were the ratios (NAA/Cr, NAA/Cho, NAA/ (Cho + Cr), Cho/Cr) of the ^1^H-MRS metabolites of the bilateral temporal poles at 1.5T and 3.0T. The data were grouped according to different magnetic field strengths and subjects’ characteristics. The Mann-Whitney U Test model was used for the 1.5T TLE group vs. 1.5T HCs, and 3.0T TLE group vs. 3.0T HCs. Each healthy volunteer was scanned using both 1.5T and 3.0T scanners, providing a set of paired and correlated data. Therefore, the Pair T-Test was used to compare 3.0T HCs with 1.5T HCs. The difference is considered statistically significant if p < 0.05. The power analysis was used to compare diagnostic capability differences between 1.5T and 3.0T scanners, and the probability of error, whose value depends on the significance criterion (α), the sample size (N), and the population effect size (ES) [23] was assessed by the G*Power (version 3.1.9.7). As shown below [24], the power value can be calculated:

1 $$\begin{array}{c}power=\text{Pr}\left(\text{r}\text{e}\text{j}\text{e}\text{c}\text{t} {H}_{0}|{H}_{1}\text{i}\text{s} \text{t}\text{r}\text{u}\text{e}\right)=1-\beta ,\end{array}$$

where H_0_ means the null hypothesis, H_1_ means the alternative hypothesis, and β means the probability of the error. The smaller power value suggests that β value is larger, and thus, the probability of the misjudgment increases, i.e. it is misjudged, even though the metabolite ratio is statistically different between 1.5T and 3.0T.

## Results

**Fig. 3:**
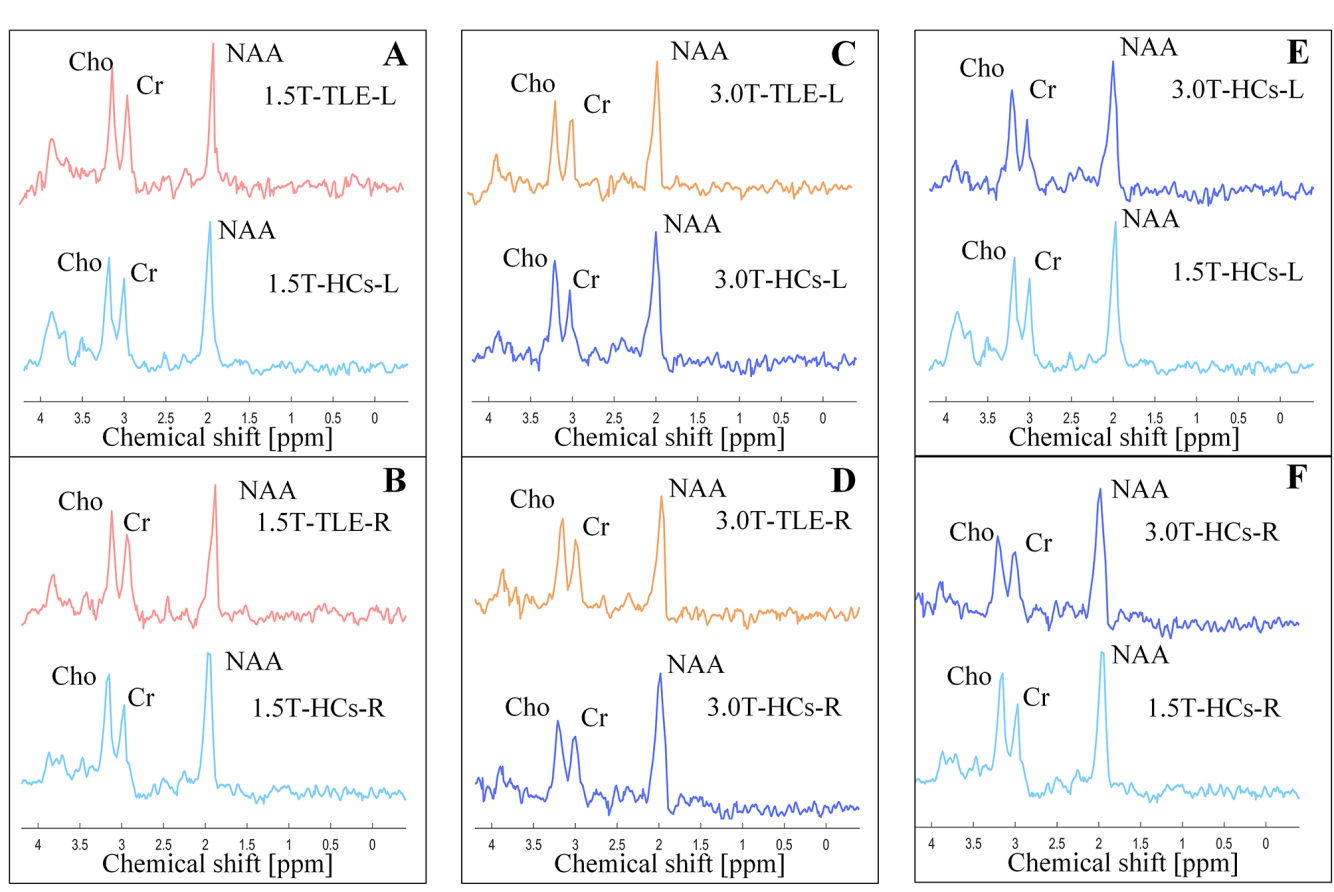
Comparison of temporal lobe MRS with different data. (**A**) Left and (**B**) right temporal poles of [TLE group vs. HCs] with 1.5T scanner. (**C**) Left and (**D**) right temporal poles of [TLE group vs. HCs] with 3.0T scanner. (E) Left and (F) right temporal poles of HCs with [1.5T vs. 3.0T] scanners. Note: L: left temporal pole, R: right temporal pole. MRS: magnetic resonance spectroscopy

Four controlled experiments were designed as followed: (1) 1.5T TLE group vs. 1.5T HCs; (2) 3.0T TLE group vs. 3.0T HCs; (3) the power analysis between the 3.0T scanner and 1.5T scanner based on the statistical test of the TLE and HCs; and (4) 3.0T HCs vs. 1.5T HCs. The comparisons of the spectra obtained from the control experiments were shown in Fig. 3.

### 1.5T TLE group vs. 1.5T HCs

**Fig. 4:**
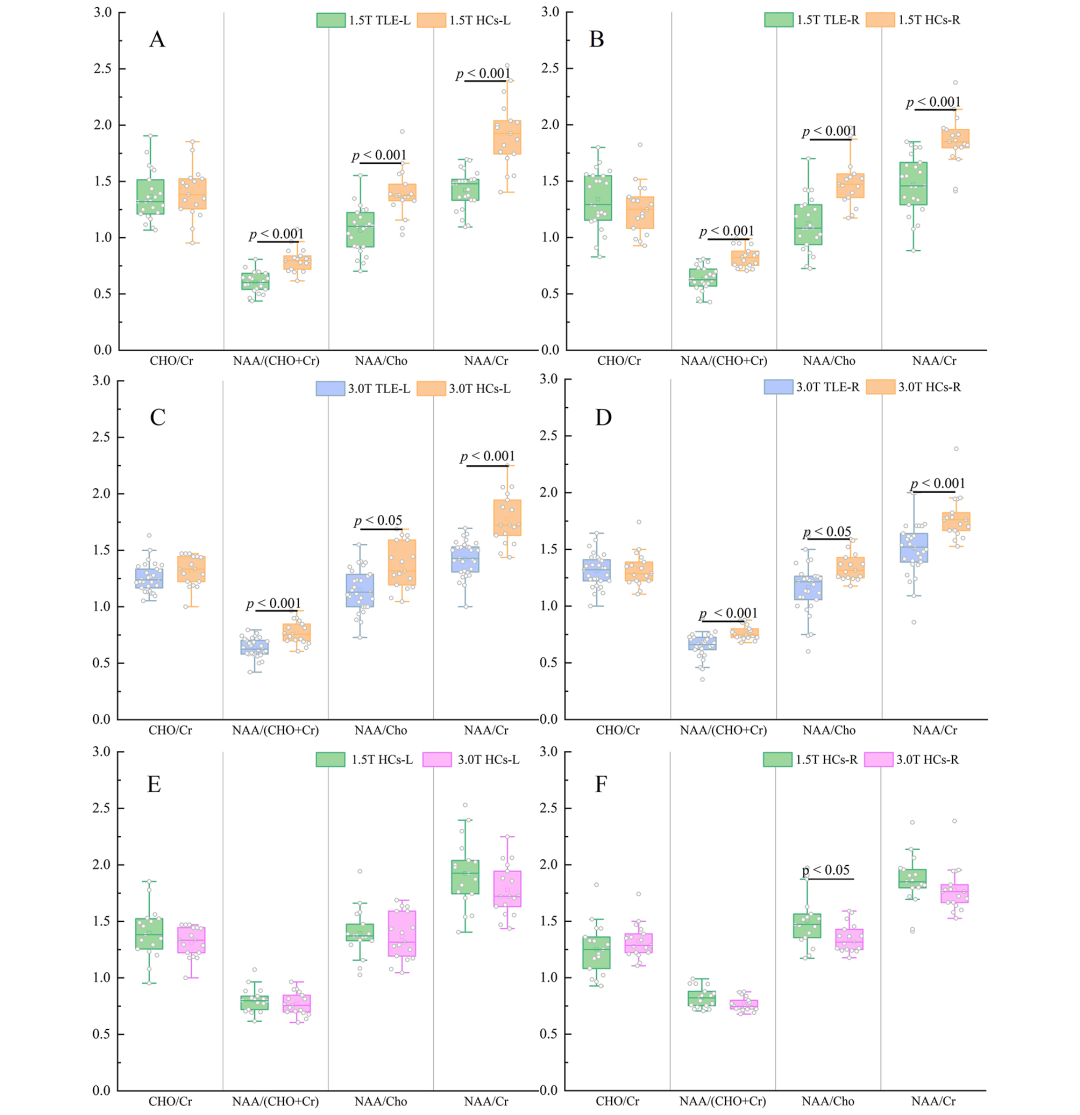
Box plots of the ratios of four metabolites in control experiments. (**A**) and (**B**) show the comparisons of the metabolite concentration ratios between the TLE group and HCs with a 1.5T scanner. (**C**) and (**D**) show the comparisons of the metabolite concentration ratios between the TLE group and HCs with a 3.0T scanner. (**E**) and (**F**) are comparisons of the ratios of metabolite concentrations of HCs at different field strengths. L: left temporal pole. R: right temporal pole

Compared with the HCs (Table 2), the ratios of the three metabolites (NAA/Cr, NAA/Cho, NAA/ (Cho + Cr)) are lower in the TLE group in the bilateral temporal poles with the 1.5T scanner. As shown in Fig. 4(A) and Table 2, for the left temporal pole, NAA/Cr, NAA/Cho and NAA/ (Cho + Cr)) ratios of the TLE group, compared with those of HCs, were statistically significant (p < 0.05), while Cho/Cr was not statistically different. The results are the same for the right temporal pole (Fig. 4(B) and Table 2). Therefore, the NAA/Cr, NAA/Cho, and NAA/(Cho + Cr) ratios of the TLE group were statistically different compared with HCs in the bilateral temporal poles.

**Table 2:** Comparison between 1.5T TLE groups and 1.5T HCs

Metabolite ratio	Left temporal pole			Right temporal pole		
	TLE	HCs	P value	TLE	HCs	P value
NAA/Cr	1.423 ± 0.173	1.926 ± 0.305	**< 0.001**	1.469 ± 0.263	1.855 ± 0.231	**< 0.001**
NAA/Cho	1.065 ± 0.206	1.394 ± 0.217	**< 0.001**	1.125 ± 0.244	1.507 ± 0.234	**< 0.001**
NAA/(Cho + Cr)	0.606 ± 0.091	0.804 ± 0.109	**< 0.001**	0.631 ± 0.109	0.825 ± 0.091	**< 0.001**
Cho/Cr	1.366 ± 0.218	1.398 ± 0.229	0.498	1.339 ± 0.258	1.254 ± 0.230	0.265

### 3.0T TLE group vs. 3.0T HCs

The results acquired from the 3.0T scanner demonstrated that the TLE group’s NAA/Cr, NAA/Cho, NAA/(Cho + Cr), and Cho/Cr ratios were lower than those of the HCs (Table 3). In the bilateral temporal poles, NAA/Cr, NAA/Cho, and NAA/(Cho + Cr) ratios of the TLE group are statistically different from those of the HCs (Fig. 4(C)(D) and Table 3), which is consistent with the findings of the 1.5T scanner above.

**Table 3:** Comparison between 3.0T TLE and 3.0T HCs

Metabolite ratio	Left temporal pole			Right temporal pole		
	TLE	HCs	P value	TLE	HCs	P value
NAA/Cr	1.427 ± 0.159	1.779 ± 0.229	**< 0.001**	1.507 ± 0.242	1.779 ± 0.201	**< 0.001**
NAA/Cho	1.149 ± 0.191	1.365 ± 0.207	**< 0.050**	1.168 ± 0.241	1.351 ± 0.126	**< 0.050**
NAA/(Cho + Cr)	0.635 ± 0.087	0.77 ± 0.102	**< 0.001**	0.656 ± 0.118	0.765 ± 0.062	**< 0.001**
Cho/Cr	1.257 ± 0.129	1.314 ± 0.136	0.099	1.310 ± 0.150	1.323 ± 0.155	0.936

### Power analysis between TLE groups and HCs

**Fig. 5:**
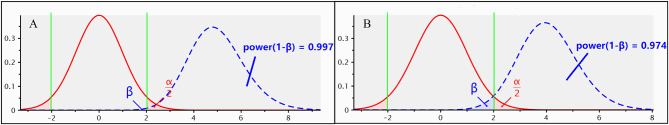
The power values and beta error of the NAA/Cr ratios. (**A**) shows the comparison between the TLE and HCs in the right temporal pole with the 1.5T scanner. (**B**) shows the comparison between the TLE and HCs in the right temporal pole with the 3.0T scanner

The power analysis was used to calculate the probability of the error of the Mann-Whitney U Test in Tables 2 and 3, and assess the utility of each metabolite ratio discriminating between TLEs and HCs on the 1.5T and 3.0T scanners. As shown in Fig. 5; Table 4, comparing 1.5T and 3.0T scanners, the power values of NAA/Cr, NAA/Cho, NAA/(Cho + Cr) and Cho/Cr were similar in the bilateral temporal poles, indicating that the four metabolite ratios had similar … abilities for TLEs and HCs on the 1.5T and 3.0 T scanners. The power values of NAA/Cr, NAA/Cho, NAA/(Cho + Cr) were greater than 0.85, namely the probability of the error was less than 15%, which means the high reliability of the results.

**Table 4:** Power analysis between TLE groups and HCs

Metabolite ratio	1.5T-L	3.0T-L	1.5T-R	3.0T-R
NAA/Cr	1.000	1.000	0.997	0.974
NAA/Cho	0.997	0.934	0.998	0.859
NAA/(Cho + Cr)	1.000	0.996	1.000	0.960
Cho/Cr	0.073	0.283	0.184	0.059

L: left temporal pole. R: right temporal pole

### 3.0T HCs vs. 1.5T HCs

Each healthy volunteer was scanned with both 1.5T and 3.0T scanners. Compared with the left temporal pole of the 1.5T HCs (Fig. 4(E) (F) and Table 5), four metabolite ratios (NAA/Cr, NAA/Cho, NAA/(Cho + Cr), Cho/Cr) of the 3.0T HCs decreased by 7.63%, 2.08%,4.22%, and 6.00%, respectively. Compared with the right temporal pole of the 1.5T HCs, NAA/Cr, NAA/Cho, and NAA/(Cho + Cr) ratios of the 3.0T HCs decreased by 4.10%, 10.35%, and 7.27%, respectively, but Cho/Cr ratio was slightly elevated (5.42%). There was no significant difference in the four metabolites ratios compared 3.0T HCs with 1.5T HCs, except for NAA/Cho in the right temporal pole (p = 0.035).

**Table 5:** Comparison between 3.0T HCs and 1.5T HCs

Metabolite ratio	Left temporal pole			Right temporal pole		
	HCs(1.5T)	HCs(3.0T)	P value	HCs(1.5T)	HCs(3.0T)	P value
NAA/Cr	1.926 ± 0.305	1.779 ± 0.229	0.100	1.855 ± 0.231	1.779 ± 0.201	0.433
NAA/Cho	1.394 ± 0.217	1.365 ± 0.207	0.721	1.507 ± 0.234	1.351 ± 0.126	0.035
NAA/(Cho + Cr)	0.804 ± 0.109	0.77 ± 0.102	0.379	0.825 ± 0.091	0.765 ± 0.062	0.080
Cho/Cr	1.398 ± 0.229	1.314 ± 0.136	0.258	1.254 ± 0.220	1.322 ± 0.155	0.349

## Discussion

Recently, MRS has been extensively studied for epilepsy, including neuronal damage in mesial TLE [25], searching for biomarkers to detect epileptogenic zone in non-lesional focal impaired awareness epilepsy [26], change of GABA level in hippocampus and anterior cingulate cortex [13], and discuss the differences in brain metabolic between TLE and organic non-epileptics [10]. To our knowledge, no other work has involved the evaluation of diagnostic utility for TLE on different field strengths.

In this work, these three metabolites NAA, Cr, and Cho were calculated as concentration ratios to evaluate the diagnostic utility of 1.5T and 3.0T ^1^H MRS for TLE. According to our findings, the TLE group’s NAA/Cr, NAA/Cho, and NAA/(Cho + Cr) ratios were lower than those of HCs. This observation was consistent with the findings of earlier studies [27]. One potential explanation is the increased cell density resulting from neuron loss or metabolic damage [10, 17, 28]. Other studies have shown that NAA concentrations are reduced in TLE. Low NAA may reflect degree of discharge from temporal lobe seizures. More important, the same statistical characteristics were discovered in the bilateral temporal poles whether 1.5T or 3.0T scanner was used.

The Mann-Whitney U Test was used to compare the two experiment results shown in Tables 2 and 3, which demonstrated that three metabolic ratios (NAA/Cr, NAA/Cho, and NAA/(Cho + Cr)) had the same statistically significant differences at both 1.5T and 3.0T. Thus, these metabolite ratios might be used as potential biomarkers to identify TLEs from HCs. Therefore, these metabolite ratios can show significant differences no matter with the 1.5T or 3.0T scanner, which may indicate that 1.5T and 3.0T have similar diagnostic efficacy for TLE.

The power analysis was used to evaluate metabolites’ ability to distinguish the TLE from the HCs as well as both 1.5T and 3.0T scanners’ diagnostic performances, i.e. sensitivity and reproducibility. Experimental results show that, the power values of NAA/Cr, NAA/Cho, NAA/(Cho + Cr) and Cho/Cr are close whether on the 1.5T or the 3.0T scanner (Fig. 5; Table 4), and the four metabolite ratios have similar ability to distinguish TLE from HC, which denotes 1.5T and 3.0T scanners have similar sensitivities and reproducibility on metabolite detection with the validation of the results of the Mann-Whitney U Test.

To reduce the influence of different subjects on the experimental results, Paired T-test (Table 5) was used to analyse the metabolite ratios of each same volunteer at 1.5T and 3.0T scanners. The results show no significant difference in metabolite ratios between the 1.5T and 3.0T scanners, whether in the left or right temporal lobe, except for NAA/Cho in the right temporal pole (p < 0.05). This suggests that 1.5T and 3.0T scanners may have similar diagnostic efficacy for HCs. There was just one metabolite ratio, NAA/Cho, that differed statistically (p < 0.05) between HCs when different field strengths are compared. There might be several reasons for the abnormal value, such as insufficient healthy volunteers, differences in manual operations during scanning.

MRS studies mainly focused on the metabolites of NAA, Cr, and Cho [10] and gradually shifted to glutamate (Glu) and gamma-aminobutyric acid (GABA) recently [29, 30]. Studies showed that the mechanism for epileptogenesis involves an imbalance between excitatory and inhibitory processes [25, 30]. This imbalance is probably associated with a higher level of extracellular Glu in the brain and a decrease in GABA concentration, leading to excitotoxicity, seizures, and cell damage [29, 30].

This work has some limitations. First, only four metabolic ratios were included in the analysis, which may be not enough. Other metabolites, such as Glu and GABA, are also important potential biomarkers of epilepsy [29]. Second, the study of NAA/Cho ought to be paid more attention if more samples can be included. Finally, studies have shown that 7 T MRS can achieve higher spectral resolution and signal-to-noise ratio, which helps to distinguish epilepsy metabolites [29]. However, this paper does not offer a profound study about the influence of the signal-to-noise ratio and resolution at different field strengths [31, 32], instead focusing on the comparison of the abilities of 1.5T and 3.0T scanners to diagnose TLE using ^1^H-MRS metabolite concentrations.

## Conclusions

To evaluation of the diagnostic utility on 1.5T and 3.0T ^1^H magnetic resonance spectroscopy for TLE, 4 controlled experiments were designed. The results showed that potential biomarkers (NAA/Cr, NAA/Cho, NAA/(Cho + Cr)) had the same efficacy using the 1.5T and 3.0T scanners for distinguishing the TLE from HCs in the bilateral temporal lobes. Thus, both 1.5T and 3.0T scanners may have comparable potential in distinguishing TLEs from HCs when ^1^H-MRS is used to identify patients with TLE.

## Data Availability

The datasets generated and/or analysed during the current study are not publicly available due to [REASON WHY DATA ARE NOT PUBLIC] but are available from the corresponding author on reasonable request.

## List of Abbreviations

^1^H-MRS: ^1^H magnetic resonance spectroscopy
TLE: Temporal lobe epilepsy
HCs: Healthy control volunteers
NAA: N-acetylaspartate
Cr: Creatine
Cho: Choline
V-EEG: Video electroencephalogram
MRI: Magnetic resonance imaging
SD: Standard Deviation
ES: Effect size
